# RETRACTION: Surgical Site Infection Following Operative Treatment of Open Fracture: Incidence and Prognostic Risk Factors

**DOI:** 10.1111/iwj.70640

**Published:** 2025-04-23

## Abstract

**RETRACTION:** HuQ. , ZhaoY. , SunB. , QiW. , and ShiP. , “Surgical Site Infection Following Operative Treatment of Open Fracture: Incidence and Prognostic Risk Factors,” International Wound Journal 17, no. 3 (2020): 708–715, 10.1111/iwj.13330.32068337 PMC7949428

The above article, published online on 18 February 2020, in Wiley Online Library (http://onlinelibrary.wiley.com/), has been retracted by agreement between the journal Editor in Chief, Professor Keith Harding; and John Wiley & Sons Ltd. Following an investigation by the publisher, all parties have concluded that this article was accepted solely on the basis of a compromised peer review process. The editors have therefore decided to retract the article. The authors did not respond to our notice regarding the retraction.

**RETRACTION:** Q. Hu , Y. Zhao , B. Sun , W. Qi , and P. Shi , “Surgical Site Infection Following Operative Treatment of Open Fracture: Incidence and Prognostic Risk Factors,” International Wound Journal 17, no. 3 (2020): 708–715, 10.1111/iwj.13330.32068337 PMC7949428

The above article, published online on 18 February 2020, in Wiley Online Library (http://onlinelibrary.wiley.com/), has been retracted by agreement between the journal Editor in Chief, Professor Keith Harding; and John Wiley & Sons Ltd. Following an investigation by the publisher, all parties have concluded that this article was accepted solely on the basis of a compromised peer review process. The editors have therefore decided to retract the article. The authors did not respond to our notice regarding the retraction.

